# Computational Nosology and Precision Psychiatry

**DOI:** 10.1162/CPSY_a_00001

**Published:** 2017-10-01

**Authors:** Karl J. Friston, A. David Redish, Joshua A. Gordon

**Affiliations:** 1 The Wellcome Trust Centre for Neuroimaging, Institute of Neurology, UCL, London WC1N 3BG, UK; 2 Department of Neuroscience, University of Minnesota, Minneapolis, MN 55455; 3 Department of Psychiatry, Columbia University, New York, NY 10032; 4 Director: National Institute of Mental Health (NIMH), Bethesda MD 20814

**Keywords:** nosology, psychiatry, psychopathology, pathophysiology, Bayesian, model selection, dynamics, therapy

## Abstract

This article provides an illustrative treatment of psychiatric morbidity that offers an alternative to the standard nosological model in psychiatry. It considers what would happen if we treated diagnostic categories not as *causes* of signs and symptoms, but as diagnostic *consequences* of psychopathology and pathophysiology. This reformulation (of the standard nosological model) opens the door to a more natural description of how patients present—and of their likely responses to therapeutic interventions. In brief, we describe a model that generates symptoms, signs, and diagnostic outcomes from latent psychopathological states. In turn, psychopathology is caused by pathophysiological processes that are perturbed by (etiological) causes such as predisposing factors, life events, and therapeutic interventions. The key advantages of this nosological formulation include (i) the formal integration of diagnostic (e.g., DSM) categories and latent psychopathological constructs (e.g., the dimensions of the Research Domain Criteria); (ii) the provision of a hypothesis or model space that accommodates formal, evidence-based hypothesis testing (using Bayesian model comparison); and (iii) the ability to predict therapeutic responses (using a posterior predictive density), as in precision medicine. These and other advantages are largely promissory at present: The purpose of this article is to show what might be possible, through the use of idealized simulations.

## INTRODUCTION

In the summer of 2015, a group of psychiatrists and computational neuroscientists convened under the auspices of an Ernst Strüngmann Forum on Computational Psychiatry (Redish & Gordon, 2016). Our brief was to consider What Can Theoretical Neuroscience and Psychiatry Teach Each Other? One of the key issues addressed by this group was the status of psychiatric nosology and how it might be informed by advances in computational neurobiology (Montague, Dolan, Friston, & Dayan, 2012; Redish & Johnson, 2007; Wang & Krystal, 2014). This article illustrates our conclusions, using simulated case studies of nosology, diagnosis, and prognosis. The original material on which this article is based can be found in Friston (2016).

Our starting point was realizing that diagnostic categories are not the causes of psychopathology—they are (observable) consequences. Although rather obvious in hindsight, this came as something of a revelation, largely because it exposed the missing link between the putative causes of psychiatric illness (e.g., genetic predisposition, environmental stressors, or iatrogenic outcomes) and the consequences, as observed by clinicians (e.g., symptoms, signs, and crucially, diagnostic outcome). See also Borsboom and Cramer (2013) for a thoughtful discussion. In what follows, we briefly rehearse the ideas—borrowed from computational neurobiology—that we hoped might close this gap.

The principal contribution of a formal or computational approach to nosology rests on the notion of a *generative model*. A generative model generates consequences from causes—in our case, symptoms, signs, and diagnoses from the underlying psychopathology and pathophysiology. Generally, the models are state-space models that describe the dynamics and trajectories in a space of latent (e.g., pathophysiological) states. These states are latent, or hidden from direct observation, and are only expressed in terms of measurable consequences, such as symptoms and signs. The utility of a generative model lies in its ability to infer latent states from observed outcomes and, possibly more importantly, the opportunity the modeling provides to assess the evidence for one model relative to others, given a set of measurements. This is known as (Bayesian) *model comparison*, where each model’s evidence is simply the probability of any sequence of observations under that particular model (Stephan, Penny, Daunizeau, Moran, & Friston, 2009). To assess the evidence for a particular model, it is necessary to fit the model to observed data—a procedure known as *model inversion*. This is because the mapping from causes to consequences is inverted, so as to map from consequences to causes—for example, inferring the pathophysiology from the symptoms. Furthermore, having inverted a model—by optimizing its parameters to maximize the model evidence—one can then simulate or predict new outcomes in the future by using something called the *posterior predictive density*. Heuristically, this is the technology behind weather forecasts, in which the generative model is a detailed state-space model of meteorological dynamics (Young, 2002). Can we conceive of an equivalent to meteorology within psychiatry?

In the past decade, considerable advances have been made in using state-space models of distributed neuronal processes to understand (context-sensitive) connectivity and functional architectures in the brain. This is known as *dynamic causal modeling*, which has been the approach adopted by a significant number of articles in the imaging neuroscience literature (Daunizeau, David, & Stephan, 2011; Friston, Harrison, & Penny, 2003). In what follows, we apply exactly the same principles to the problem of modeling the causes of nosological outcomes in psychiatry. Indeed, all the examples below use standard Bayesian model inversion schemes that are available in freely available academic software: the simulations described below can be reproduced by downloading the SPM software (www.fil.ion.ucl.ac.uk/spm/) and running the MATLAB script DEM_demo_ontology.m. The dynamic causal modeling of psychopathology can, in principle, offer a number of advantages over the standard nosological model. As we noted above, in the abstract, these include (i) the formal integration of diagnostic (e.g., the *Diagnostic and Statistical Manual of Mental Disorders* [DSM]) categories and latent psychopathological constructs (e.g., the dimensions of the Research Domain Criteria [RDoC]; Stephan & Mathys, 2014); (ii) the provision of a hypothesis or model space that accommodates formal, evidence-based hypothesis testing (Krystal & State, 2014); and (iii) the ability to predict therapeutic responses (using a posterior predictive density). Crucially, by adopting a dynamic modeling approach, one can properly accommodate the personal histories and trajectories of individual patients when determining (and predicting) the course of their illness. This was a key aspect of our group discussions—and something that is currently underemphasized in standard nosology.

Our overall approach to nosology (and the advantages it promises) may seem rather abstract and grandiose. What follows is therefore offered as a prospectus for future discussions about nosology and the potential for individualized or precision psychiatry in the future. Our purpose is to illustrate what could be possible, in an idealized world, if we could use (dense) clinical data to optimize generative models of psychopathology, in the context of high-throughput personalized medicine (Richesson, Sun, Pathak, Kho, & Denny, 2016). The data we have in mind speak to the new era of digital health—for example, data from wearable (e.g., pedometer) sensors and mobile-application-based tools supervised by the patient or clinician (Hollis et al., 2015; Martinez & Valstar, 2016). Whether or not this will be possible with current data is an open question. In short, this article offers a formal sketch of what a computational nosology could look like.

The article comprises three sections. The first describes a model that generates symptomatic and diagnostic outcomes from latent (pathophysiological and psychopathological) causes. This particular model should not be taken too seriously: it is just used to show that a formal approach to nosology forces one to think carefully about the known and unknown variables in psychiatric processes, and how they may influence each other. The next section considers the ability to use ratings of symptoms and signs (and diagnosis) to estimate or infer their latent causes. This is a necessary prelude to the notion of model comparison that is briefly illustrated in this section. Finally, we consider prognosis and prediction by using the generative model to predict the outcome of a (simulated) schizoaffective process—and its response to treatment.

## GENERATIVE MODELS FOR PSYCHIATRIC MORBIDITY

This section introduces the general form of generative (dynamic causal) models for psychiatric morbidity, along with a particular example that will be used to illustrate model inversion, model selection, and prediction in subsequent sections. As we noted above, a generative model generates consequences from causes. The basic form assumed here starts with the (known) causes of psychiatric illness, such as genetic or environmental predispositions and therapeutic interventions. These factors act upon pathophysiological states—such as aberrant dopamine receptor availability or glucocorticoid receptor function—to alter their trajectories over a course of weeks to months. These latent pathophysiological states then determine a psychopathology, cast in terms of latent cognitive, emotional, or behavioral function (e.g., low mood, psychomotor poverty, thought disorder, etc.). Psychopathological states correspond to the constructs underlying such methods as RDoC (Kaufman, Gelernter, Hudziak, Tyrka, & Coplan, 2015) and clinical brain profiling (Peled, 2009). Finally, the psychopathological states generate measured symptoms using, for example, standardized instruments (e.g., the Positive and Negative Symptoms of Schizophrenia Scale [PANSS; Kay, 1990], Beck Depression Inventory, mini mental state, etc.) or diagnostic outcomes (e.g., schizophrenia, major affective disorder, or schizoaffective disorder).

Note that in this setup, a diagnosis represents an outcome provided by a clinician. In other words, the symptoms, signs, and diagnosis have a common cause, and the diagnostic categorization provides a useful summary outcome that integrates aspects of psychopathology that may not be covered explicitly by standardized symptom ratings or particular signs (psychomotor poverty, electroencephalographic abnormalities, abnormal dexamethasone suppression, etc.).

**Figure 1.**
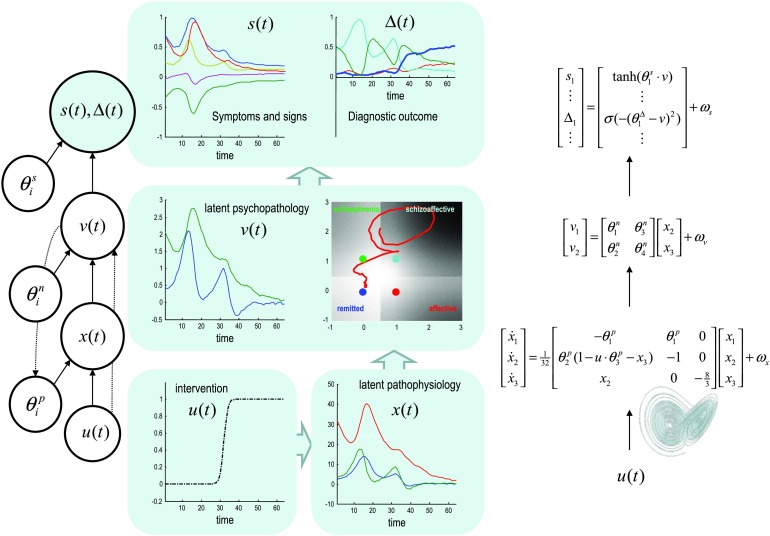
**Schematic of a generative model for psychiatric morbidity.** The model is shown in terms of a (probabilistic) graphical model on the left. In this format, the quantities in white circles correspond to random variables that include (unknown) parameters on the left and hidden or latent states on the right. The arrows denote conditional dependencies and describe the influences among latent variables that generate the observations or outcomes in the cyan circle at the top. Here, the outcomes are clinical symptom scores and a differential diagnosis, measured as a probability distribution over diagnostic classifications. The only difference between these outcomes is that the diagnostic probabilities are constrained to be nonnegative and to sum to 1 (so that they can be treated like a proper probability distribution over a differential diagnosis). The outcomes are generated as functions of psychopathological states that, themselves, are mixtures of pathophysiological states. Finally, the pathophysiological states are perturbed by inputs (such as therapeutic interventions). An example of the form of the conditional dependencies is provided on the right, in terms of functions and random (Gaussian) fluctuations. In this example, a single therapeutic intervention enters the dynamics (or equations of motion) governing the evolution of the pathophysiological states. Here, the therapeutic intervention changes the influence of the second physiological state on the first, where this coupling itself is state-dependent (and changes with the third state). These dynamics are based on a Lorenz attractor (illustrated by the insert in the lower corner). The psychopathological states are generated as a linear mixture of the last two physiological states. In turn, the psychopathological variables are mapped to clinical outcomes through sigmoid functions (to generate symptom scores) and a softmax function of diagnostic potential (to generate a differential diagnosis). The diagnostic potential is based on the proximity of the psychopathological state to locations representing diagnostic categories. The panels in the middle column illustrate a particular realization of this generative model over 64 time bins (i.e., weekly assessments). The lower panels in this sequence show the therapeutic input (starting at 32 weeks) and the dynamic responses of the three pathophysiological states. The ensuing psychopathology is shown both as a function of time and as a trajectory in state-space in the left and right middle panels, respectively. The state-space of psychopathology contains the locations associated with diagnostic categories (colored dots), which determine the diagnostic classification that tiles the state-space (four regions of gray, weighted by the entropy of the probability distribution over diagnoses). This example includes four diagnostic categories (schizophrenia, schizoaffective and affective disorders, and a state of remission). The symptoms and differential diagnosis generated by this trajectory are shown in the upper panels on the left and right, respectively. In this and subsequent simulations, the initial (physiological) states are [8, 10, 32]; the parameters for the symptom scores are sampled from a unit Gaussian distribution, and the remaining parameters are shown in Figure 4.

This formulation of psychiatric nosology is largely common sense and reiterates what most people would understand about psychiatric disorders. However, can this understanding be articulated formally, in a way that can be used to make quantitative predictions and to test competing etiological hypotheses? This is where a formal nosology or generative model comes into play. The first step is to construct a *graphical model*of dependencies among the variables that generate the measurable outcomes. Figure 1 (left panel) shows a graphical model that summarizes the probabilistic dependencies among etiological causes *u*(*t*), pathophysiological states *x*(*t*), psychopathology *v*(*t*), and the symptoms and diagnosis [*s*(*t*), Δ(*t*)]. In this format, the variables in white circles correspond to latent states that are hidden from direct observation, whereas the observable outcomes are in the cyan circle.

Probabilistic dependencies are denoted by arrows that entail (time-invariant) parameters *θ* = (*θ*^*s*^,*θ*^*n*^,*θ*^*p*^). This formulation clarifies the roles of different quantities and makes their interdependencies explicit. For example, a diagnostic classification at a particular time would be an outcome variable, whereas a patient’s drug history would be an etiological cause that influenced the pathophysiology. Having established the form of the graphical model, it is now necessary to specify the nature of the dependencies within and among the latent variables. An example is provided in the right panel of Figure 1 and illustrated graphically in the middle panels. This model will be described in the following section.

### A Generative (Dynamiz Causal) Model

This example of a generative model is deliberately very simple, restricting itself to modeling a limited differential diagnosis that includes schizophrenia, affective disorder, schizoaffective disorder, and a remitted state. These diagnostic outcomes are accompanied by six symptom scores that have been normalized to lie between plus and minus one. Furthermore, we consider only a limited number of exogenous causes and latent variables: Specifically, a single therapeutic cause perturbs the evolution of three physiological states, from which two psychopathological states are derived. At any one time, the location in the psychopathological state-space determines a symptom profile (through a linear mixture of psychopathological states that is passed through a sigmoid function). Diagnostic outcomes are, here, encoded by a probability profile over the differential diagnosis (i.e., the relative confidence that a clinician places in the differential diagnoses afforded by some accepted nosological scheme; e.g., DSM or ICD). In this model, the probability of any diagnosis corresponds to its relative proximity to the current point in a psychopathological state-space. In other words, as the disorder progresses, a trajectory is traced out in a two-dimensional psychopathological state-space, where, at any one time, the prevalent diagnosis is determined by the diagnosis point the current state is closest to. Technically, this has been modeled by a softmax function of diagnostic potential, which is the (negative) Euclidean distance between the current state and the locations associated with the different diagnostic categories (encoded by $\theta_{i}^{\Delta }$—the color dots in Figure 1).

The trajectory of psychopathology is determined by the corresponding trajectory through a pathophysiological state-space that has its own dynamics. These are encoded by equations of motion or flow—based, in this example, on a Lorenz attractor (Lorenz, 1963). See van de Leemput et al. (2014) for an example of modeling mood using a (simplified) Lotka–Volterra system. The Lorenz form for the dynamics or kinetics of pathophysiology is a somewhat arbitrary choice, but it provides a ubiquitous model of chaotic dynamics in the physical (Poland, 1993) and biological (de Boer & Perelson, 1991) sciences. Interestingly, it arose from the modeling of convection dynamics, which speaks to the analogy between the present modeling proposal and weather forecasting. In this setting, the ensuing dynamics can be regarded as a canonical form for nonlinear coupled processes that might underlie the pathophysiology in psychosis. It has a canonical form because, as we see in Figure 1 (on the right), one can regard the parameters as specifying coupling coefficients or connections that mediate the influence of one physiological state on the others. Crucially, some of these connections are state-dependent. This is important because it means that one can model fluctuations in pathophysiology in terms of self-organized (chaotic) dynamics that have an underlying attracting set. In other words, the Lorenz form provides a canonical summary of slow fluctuations in neuronal (or hormonal) states that show homoeostatic or allostatic tendencies (e.g., Leyton & Vezina, 2014; Misiak, Frydecka, Zawadzki, Krefft, & Kiejna, 2014; Oglodek, Szota, Just, Mos, & Araszkiewicz, 2014; Pettorruso et al., 2014).

The equations on the right of Figure 1 all include random fluctuations. These fluctuations render the generative model a probabilistic statement about how a number of variables could influence each other. Here, the random fluctuations can be regarded as observation noise (when assessing symptoms) and as random fluctuations or perturbations to psychological or physiological processing. These fluctuations stand in for transient changes in daily experiences and neurophysiological processes that are not predicted by the slower dynamics of psychological and physiological states (modeled by the equations of motion). In this article, these random fluctuations are smooth processes with a Gaussian correlation function and a correlation length of half an assessment interval (i.e., a few days).

We make no pretense that any of these states map in a simple way onto the physiological variables; rather, they stand in for mixtures^1^ of physiological variables that have relatively simple dynamics. The existence of mixtures is assured by technical theorems such as the center manifold theorem and the slaving principle in physics (Carr, 1981; Davis, 2006; Frank, 2004; Haken, 1983). Crucially, we have constructed this generative model such that the therapeutic intervention changes the state-dependent coupling between the first and second pathophysiological states (in fluid dynamics, this control parameter is known as a *Rayleigh number* and reflects the degree of turbulent flow). This means that we can regard the intervention as a pharmacotherapy that changes the coupling between different neuronal (or hormonal) systems—for instance, the influence of an atypical antipsychotic (Hrdlicka & Dudova, 2015) on the dopaminergic and 5HT receptor function responsible for both monoaminergic tone in the ventral striatum and serotoninergic projections from the amygdala to the paraventricular nucleus (Muzerelle, Scotto-Lomassese, Bernard, Soiza-Reilly, & Gaspar, 2014; Wieland et al., 2015). Furthermore, we have introduced a parameter $\theta_{3}^{p}$ that determines the sensitivity to the intervention, which may be important in determining a patient’s responsiveness to therapy (Brennan, 2014). Note that although we have portrayed the intervention as therapeutic, we could have treated it as some known change in life circumstances—for example, a life event (Kendler & Karkowski-Shuman, 1997) that has a persistent effect on neuroendocrine function or neurophysiology.

The middle panels of Figure 1 provide an illustration of how a patient might present over time under this particular model. Imagine that we wanted to model six symptom scores and a probabilistic differential diagnosis over four diagnoses (schizophrenia, schizoaffective, affective, and remitted) when assessing an outpatient on a weekly basis for 64 weeks. In this illustrative example, the symptom scores could correspond to (normalized) clinical ratings from the PANSS (Kay, Fiszbein, & Opler, 1987) and the Beck Depression Inventory:

•  Delusions [PANSS: minimum score = 1, maximum score = 7]
•  Conceptual disorganization [PANSS: minimum score = 1, maximum score = 7]
•  Blunted affect [PANSS: minimum score = 1, maximum score = 7]
•  Emotional withdrawal [PANSS: minimum score = 1, maximum score = 7]
•  Anxiety [PANSS: minimum score = 1, maximum score = 7]
•  Beck Depression Inventory [BDI: minimum score = 0, maximum score = 63]

with diagnoses according to the *ICD-10 Classification of Mental and Behavioural Disorders: Diagnostic Criteria for Research* (World Health Organization, 1993):

•  F20 Schizophrenia
•  F25 Schizoaffective disorder
•  F32 Depressive episode
•  No symptoms

A therapeutic intervention—say, an atypical antipsychotic—is introduced at 32 weeks, and we want to model the response. This therapeutic input is shown in the lower left panel as a dot-dashed line, and it affects the evolution of physiological states according to the equations of motion on the right. These equations generate chaotic fluctuations in (three) pathological states, shown at lower right in the middle group of panels. Two of these states are then mixed to produce a trajectory in a psychopathological state-space. This trajectory is shown in the center panels as a function of time (left panel) and as a trajectory in state-space (right panel). In other words, the trajectory (red line) is defined by plotting the two psychopathological variables against each other. In turn, the psychopathology generates symptom scores (shown as colored lines at the upper left) and diagnostic probabilities (shown at the upper right). The relationship between the continuous (dimensional) latent space of psychopathology and the (categorical) differential diagnosis is determined by diagnostic parameters $\theta_{i}^{\Delta }$, defining the characteristic location of the *i*th diagnosis.

These locations are shown as dots in the state-space, where the blue dot corresponds to a diagnosis of remission, and the green, cyan, and red locations correspond to diagnoses of schizophrenia and of schizoaffective and affective disorders, respectively. One can see that initial oscillations between schizophrenia and schizoaffective diagnoses are subverted by the therapeutic intervention. At this point, the latent pathophysiology is drawn to its (point) attractor at zero, the most likely diagnosis becomes remission, and the symptom scores regress to their normal values of zero. In short, this models a successful intervention in a pathophysiological process that shows chaotic oscillations expressed in terms of fluctuating symptoms and differential diagnosis. We will see later that—in the absence of therapy—these chaotic oscillations would otherwise produce a relapsing–remitting progression with an ambiguous diagnosis that fluctuates between schizophrenic and schizoaffective. This intervention is formally similar to what is known anecdotally as *chaos control* (e.g., Rose, 2014). This example suggests that the goal of therapy is not so much countering the pathological deviations as the more subtle problem of suppressing chaotic or turbulent neurohormonal processes that are equipped with many self-organizing feedback mechanisms. Heuristically, the role of the clinician becomes like the captain of a ship who uses prevailing winds to navigate toward calmer waters.

This particular example is not meant to be definitive or valid in any sense. It is just one of a universe of potential models (or hypotheses) about the way that psychiatric morbidity is generated. We will return to procedures for comparing models in the next section. However, this example allows us to make a few key points about the nature of pathology and its expression. First, in any generative model of psychopathology there is a fundamental distinction between (time-invariant) parameters and (time-sensitive) states. This distinction can be regarded as the formal homologue of the distinction between *trait* and *state* abnormalities. For example, the patient illustrated above had a particular set of parameters $\theta_{i}^{p}$ determining the family of trajectories (and their attracting sets) of pathophysiology. However, simply knowing these parameters does not tell us anything about the patient’s pathological state at a particular time. To determine this, we have to infer the latent pathophysiology in terms of the current state *x*(*t*), by using model fitting or inversion. This presents a difficult (but solvable) problem, because we have to estimate both the parameters (traits) and states of a patient in order to determine that patient’s trajectory in the short term.

The second distinction this sort of model brings to the table is between parameters that are patient-specific and parameters that are conserved over the population to which the model applies. In statistical terms, this corresponds to the difference between *random* and *fixed* effects, in which patient-specific effects model random variations in traits that may reflect predisposing factors (e.g., genetic predisposition). Conversely, other parameters may be fixed over patients and determine the canonical form of nosology.

In the example above, this distinction is illustrated by the difference between parameters that are specific to each patient or pathophysiology, *θ*_*i*_^*p*^, and those that are inherent in the nosology, $\theta_{i}^{n}$. The nosological parameters define a generic mapping from pathophysiology to psychopathology that is conserved over patients. Understanding this distinction is important practically, because nosological parameters can only be estimated from group data. We will see examples of this process in the next section.

## MODEL INVERSION AND SELECTION

In this section we consider the inversion and selection of generative models based on measurable outcomes. The ultimate aim of modeling is to predict the outcomes for a particular patient. The quality of these predictions rests upon a model that is accurate and that generalizes to the sorts of patients encountered. The quality of a model is scored in terms of its evidence, given some data. However, to evaluate model evidence, one needs to be able to invert or fit the data. This means that we first have to ensure that our models can be inverted. In other words, can we recover the unknown parameters and latent variables responsible for clinical data? In what follows, we take the simulated patient above and see whether we can recover the latent states, given the (known) therapeutic input and clinical outcomes (symptoms and diagnosis). We then briefly review Bayesian model comparison and discuss its crucial role in hypothesis testing—and in elaborating a more mechanistic nosology for psychiatry in the future.

### Model Inversion and Bayesian Filtering

The problem of estimating unknown parameters and latent states from time-series data is known as *deconvolution* or *filtering* in the modeling literature. Because we have to estimate both parameters and states, this presents a *dual estimation problem* that is usually accom modated by treating parameters as very slowly fluctuating states. We will illustrate Bayesian filtering using an established procedure called *dynamic expectation maximization*(DEM). DEM was originally devised to infer latent neuronal states and the connectivity parameters generating neurophysiological signals in distributed brain networks, and it has been applied in a number of different contexts (Friston, Trujillo-Barreto, & Daunizeau, 2008). Special cases of DEM include Kalman filtering (when the states are known and the state-space model is linear).

**Figure 2.**
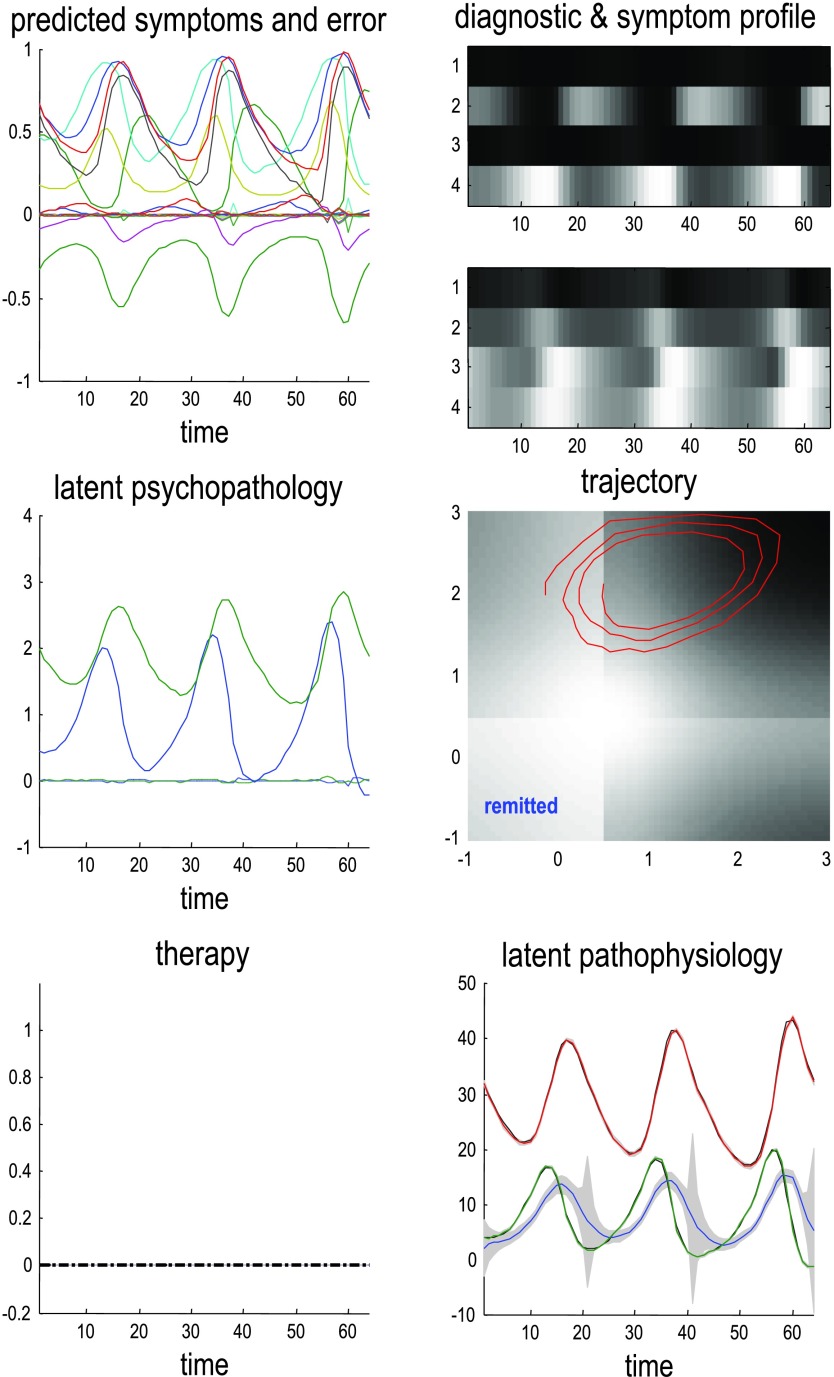
**Bayesian filtering or model inversion of simulated clinical time-series data.** This figure reports the results of model inversion using the data generated by the model described in the previous figure. In this instance, the clinical data were simulated without any therapeutic intervention (shown by the broken line in the lower left panel) over 64 weeks and are shown in a format similar to the middle panels of Figure 1. Here, the solid lines represent predictions, whereas the dotted lines represent prediction errors in terms of the clinical outcomes (upper left panel) and the latent psychopathology (middle left panel). The gray areas around the estimates of pathophysiology (lower right panel) correspond to 90% confidence intervals for the hidden states. The symptom scores and differential diagnoses are shown as a function of time (upper left) and in image format (upper right). The upper image at top right shows the changes in differential diagnosis, with the diagnosis of remission in the first (i.e., top) row, and the lower image shows the fluctuations in the first four symptom scores. Notice here that, in the absence of treatment, the chaotic fluctuations between schizophrenia and schizoaffective regimes of latent psychopathology slowly increase in amplitude.

Figure 2 shows the results of Bayesian filtering when applied to the symptom and diagnostic time series shown in the previous figure. The format is similar to the middle panels of Figure 1; however, here the colored lines correspond not to the true values generating the data, but to the estimated trajectories based on Bayesian filtering (as implemented with the MATLAB routine spm_DEM.m). In this example, we simulated a clinical progression in the absence of any therapeutic intervention (as shown by the flat line in the lower left panel). In the absence of any check on the pathophysiology, chaotic oscillations of slowly increasing amplitude emerge over a period of 64 weeks (middle right panel). The resulting fluctuations in psychopathology are shown as a function of time and as a trajectory in state-space (middle panels). In the lower right panel, the posterior expectations (the most likely trajectories) of the pathophysiological states are contained within 90% Bayesian confidence intervals (gray areas). Notice that the first pathophysiological state has greater confidence intervals and is therefore estimated with slightly less precision than the remaining two states. Interestingly, the uncertainty about the first pathophysiological state itself fluctuates, peaking at certain points in the cycle (due to the nonlinearities in the generative model). In this example, the true (not shown) and estimated values were almost identical. This is because we used very low levels of random fluctuations, modeling random effects at the levels of outcomes, psychopathology, and pathophysiology (see Figure 1).

**Figure 3.**
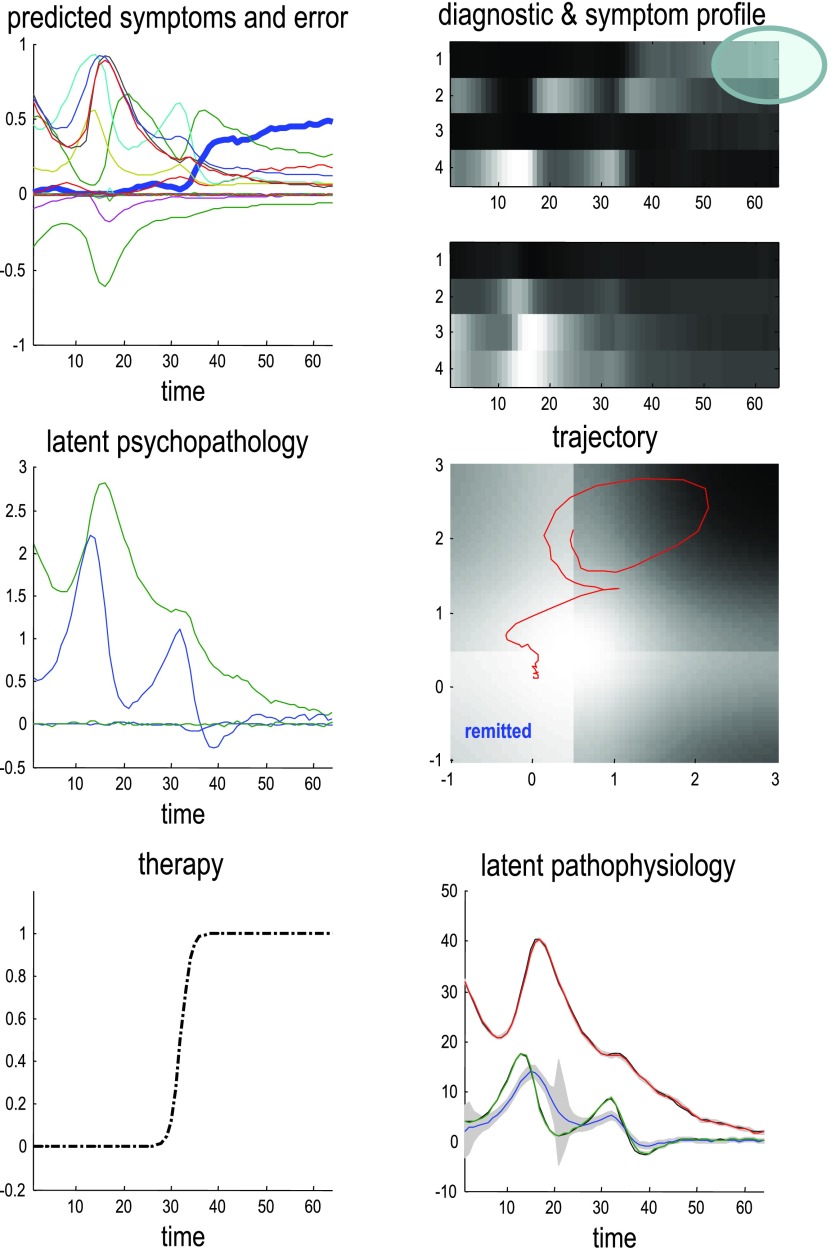

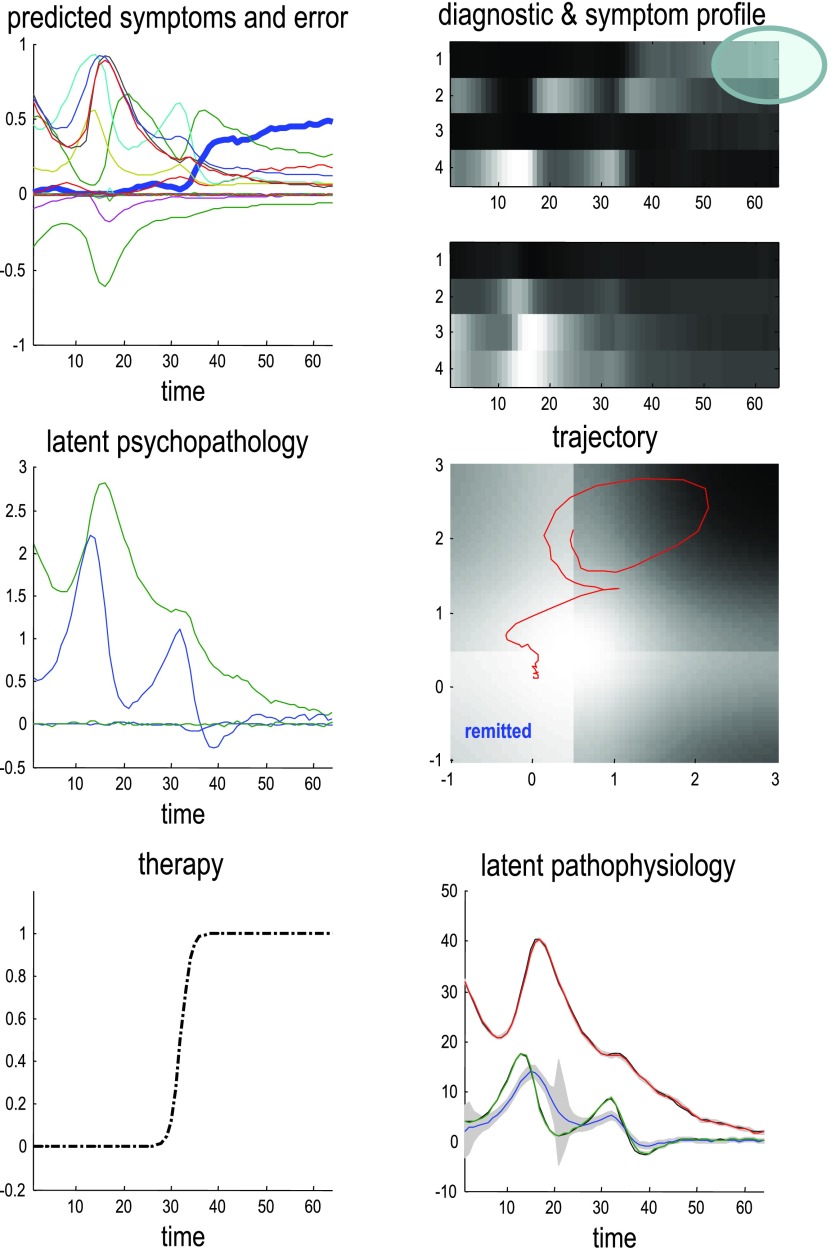
**Inferred psychopathology and pathophysiology in response to treatment.** This figure uses the same format as Figure 2. The only difference here is that we have introduced a therapeutic intervention that has destroyed the chaotic attractor, replacing it with a point attractor in the remitted regime of latent psychopathology. As a consequence, the pathophysiological variables approach zero, and the symptom scores normalize. At the same time, the most probable diagnosis becomes one of remission (solid blue line in the upper left panel—see also the cyan circle in the corresponding predictions in image format).

The upper panels show the resulting fluctuations in symptom and diagnostic scores as a function of time in graphical format (upper left panel) and in image format (upper right panel). One can see clearly that the differential diagnoses of schizophrenia and schizoaffective disorder vary every few months, reflecting an unstable and ambiguous diagnostic picture. We then repeated the filtering, but with a therapeutic intervention at 32 weeks. The simulated response and inferred latent states are shown in Figure 3. These reproduce the results of Figure 1 and show the success of the intervention—as indicated by the emergence of a remitted diagnosis as time progresses (solid blue line on the upper left and cyan circle on the upper right).

**Figure 4.**
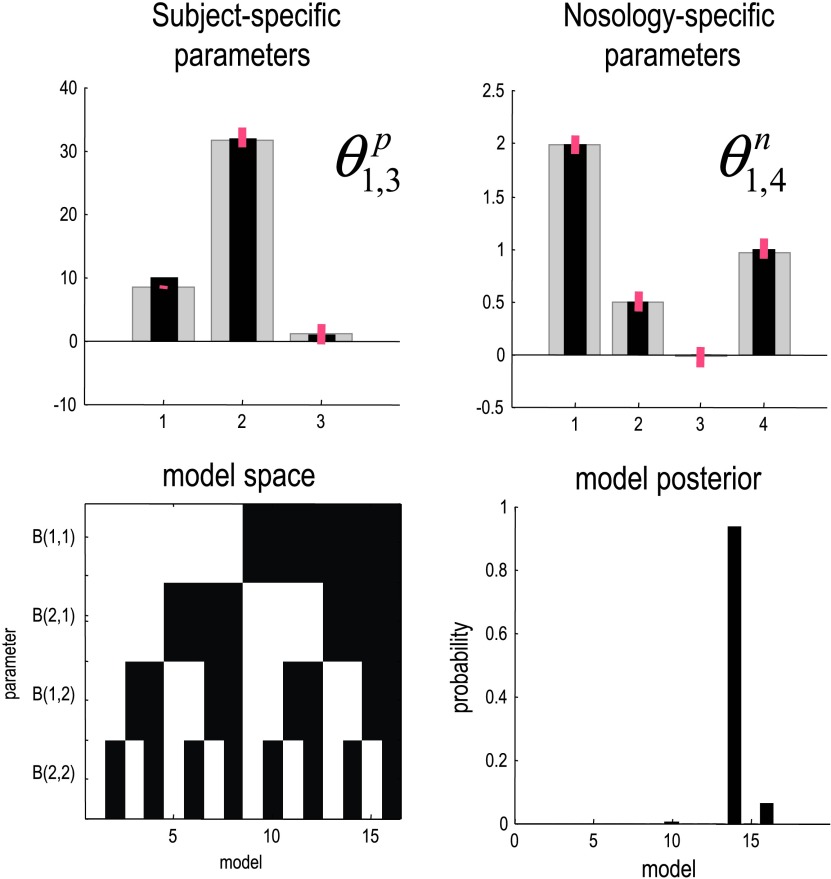
**Bayesian model identification and comparison.** This figure summarizes the results of model identification (parameter estimation) and model selection using Bayesian model reduction. The upper panels show the posterior estimates of the subject-specific (left) and nosological (right) parameters based on single-subject time series and (eight-subject) group data, respectively. The gray bars correspond to the posterior means, and the pink bars report 90% Bayesian confidence intervals. These are superimposed on black bars that correspond to the true values used to simulate the clinical data. The lower left panel shows the combinations of nosological parameters that define 16 competing models that were compared using Bayesian model reduction. This comparison entails evaluating the evidence for each model—namely, the probability of the data under each model—after having marginalized over unknown parameters and states. The model evidence is also known as the *marginal* or *integrated likelihood*. Under uninformative or flat priors over models, this also corresponds to the model posterior. The posterior probabilities over 16 models for the group data are shown at the lower right, suggesting that a model that precludes coupling between the second physiological state and the first psychopathological state (mediated by the third nosological parameter at upper right) has greater evidence than all other models.

In these illustrations, we estimated both the unknown states generating (simulated) clinical data and the patient-specific (trait) parameters governing pathophysiological dynamics. The estimated and true parameters are shown in the upper left panel of Figure 4: The estimated values are shown as gray bars (with pink 90% confidence intervals), and the true values are shown in black. The accuracy of these estimates is self-evident, with a slight overconfidence that is characteristic of the approximate Bayesian inference implicit in DEM (MacKay, 1995). Although these estimates show that, in principle, one can recover the traits and states of a particular subject at a particular time, we used the true values of the nosological parameters coupling pathophysiology to psychopathology (and generating clinical outcomes) during model inversion. One might ask, can these parameters also be estimated?

To illustrate this estimation, the upper right panel of Figure 4 reports the estimates (and 90% confidence intervals) based on an *empirical Bayesian*analysis (Kass & Steffey, 1989) of eight simulated patients (using spm_dcm_peb.m). *Empirical* Bayesian analysis refers to the hierarchical modeling of within- and between-subjects effects that may or may not be treated as random effects. In other words, empirical Bayes allows one to extend dynamic causal modeling of the subject-specific time series into group studies (Friston et al., 2016). Again, the (group mean) estimates are remarkably accurate, suggesting that, in principle, it is possible to recover parameters that are conserved over subjects. Note that the third nosological parameter has an estimated value of zero. This is important, because it leads us into the realm of Bayesian model comparison and evidence-based hypothesis testing.

### Bayesian Model Comparison and Hypothesis Testing

Above, we have seen that this sort of model can, in principle, be inverted such that underlying (latent) psychopathological and pathophysiological states can be inferred, in the context of (unknown) subject-specific parameters or traits. However, this does not mean that the model itself has any validity or will generalize to real clinical data. In other words, how do we know we have a good model?

This is a question of model comparison. In short, the best model provides an accurate explanation for the data with minimum complexity. The model evidence reflects this, because a model’s evidence is equal to accuracy minus complexity. The complexity term is important, because it ensures that models do not overfit the data—and will therefore generalize to new data. The model evidence is evaluated by marginalizing (i.e., averaging over) unknown parameters and states to provide the probability of some data under a particular model. The model here is defined in terms of the number of states (and parameters) and how they depend upon each other. A simple example of model comparison is provided in Figure 4 (lower panels).

In our model, the physiological states could influence psychopathology in a number of ways. Our model presupposes two physiological states that can influence two pathophysiological states, creating four possible dependencies that may or may not exist. This leads to 2^4^ = 16 models that cover all combinations of the nosological parameters (see the lower left panel of Figure 4). We can evaluate the evidence for each of these 16 models by inverting all 16 and evaluating the evidence, or, as illustrated here, inverting the model with all four parameters in place and computing the evidence of all the reduced models with one or more parameters missing. This is known as *Bayesian model reduction*, which is an efficient way of performing Bayesian model comparison (Friston & Penny, 2011). The results of this model comparison are shown in the lower right panel of Figure 4 and suggest that the posterior probability is much greater for Model 14 than for any of the others. In this model, the influence of the second pathophysiological state on the first psychopathological state has been removed. Removing this parameter reduces the model complexity without any loss in accuracy, and therefore increases the model evidence. We might have guessed that this was the case by inspecting the posterior density of the third nosological parameter, which mediates this model component (see the upper right panel).

This is a rather trivial example of model comparison, but it illustrates an important aspect of dynamic causal modeling: namely, the ability to test and compare different models or hypotheses. Although it is not illustrated here, one can imagine comparing models with different numbers of pathophysiological states and different forms of dynamics. One could even imagine comparing models with different graphical structures. One interesting example here would be the modeling of psychotherapeutic interventions that might influence pathophysiology through experience-dependent plasticity. This would necessitate comparing models in which a therapeutic intervention influenced psychopathology, which couples back to pathophysiology through the parameters of its dynamics. This is illustrated by the dotted arrows at the left of Figure 1.

We could speculate about many other examples: Crucial examples here involve an increasingly mechanistic interpretation of pathophysiology, in which pathophysiological states could be mapped onto neurotransmitter systems through careful (generative) modeling of electrophysiological and psychophysical measurements (Stephan & Mathys, 2014). One could also contemplate comparing models with different sorts of inputs or causes, ranging from social or environmental perturbations (e.g., traumatic events) to genetic factors (or their proxies, such as family history). Questions about whether and where genetic polymorphisms affect pathophysiology are formalized by simply comparing different generative models that accommodate effects on different states or parameters. For example, do models that include genetic biases on physiological parameters have greater evidence than models that do not?

The potential importance of model comparison should not be underestimated. We have tried to give a flavor of its potential. It is also worth noting that this field is an area of active research, with fast and improved schemes for scoring large model spaces being developed year by year (see also Viceconti, Hunter, & Hose, 2015). One can construe an exploration of model space as a greedy search through a field of competing hypotheses and a formal statement of the scientific process. This may be especially relevant for psychiatry, which deals with the special problem of integrating both physiological and psychological therapies—calling for generative models that map between these two levels of description. In the final section, we turn to the more pragmatic issue of predicting a response to treatment for an individual patient.

## PREDICTION AND PERSONALIZED PSYCHIATRY

Let us assume we have used Bayesian model comparison to optimize our generative model of psychosis and prior probability distributions over its parameters. Can we now use the model to predict the outcome of a particular intervention in a given patient? In the previous section, we saw how clinical data from a single subject could be used to estimate subject-specific parameters (traits) and states at a particular time. In fact, the parameter estimates in Figure 4 were based on the first 32 weeks of data *before any treatment began*. This means that we now have estimates of how this particular subject would respond from any physiological state, as well as the physiological state at the end of the period of assessment. Given these (posterior probability) estimates, there are several ways in which we can predict the clinical prognosis—and the responses to different treatments. The simplest way would be to sample from the posterior distribution and integrate the generative model with random fluctuations to build a probability distribution over future states. This is basically how weather forecasting is done. We will illustrate a related but simpler approach, applying Bayesian filtering to null data with zero precision; in other words, we fit time-series data that have yet to be acquired. This provides a predictive distribution over future trajectories based on the posterior estimates of the subject’s current parameters and states.

**Figure 5.**
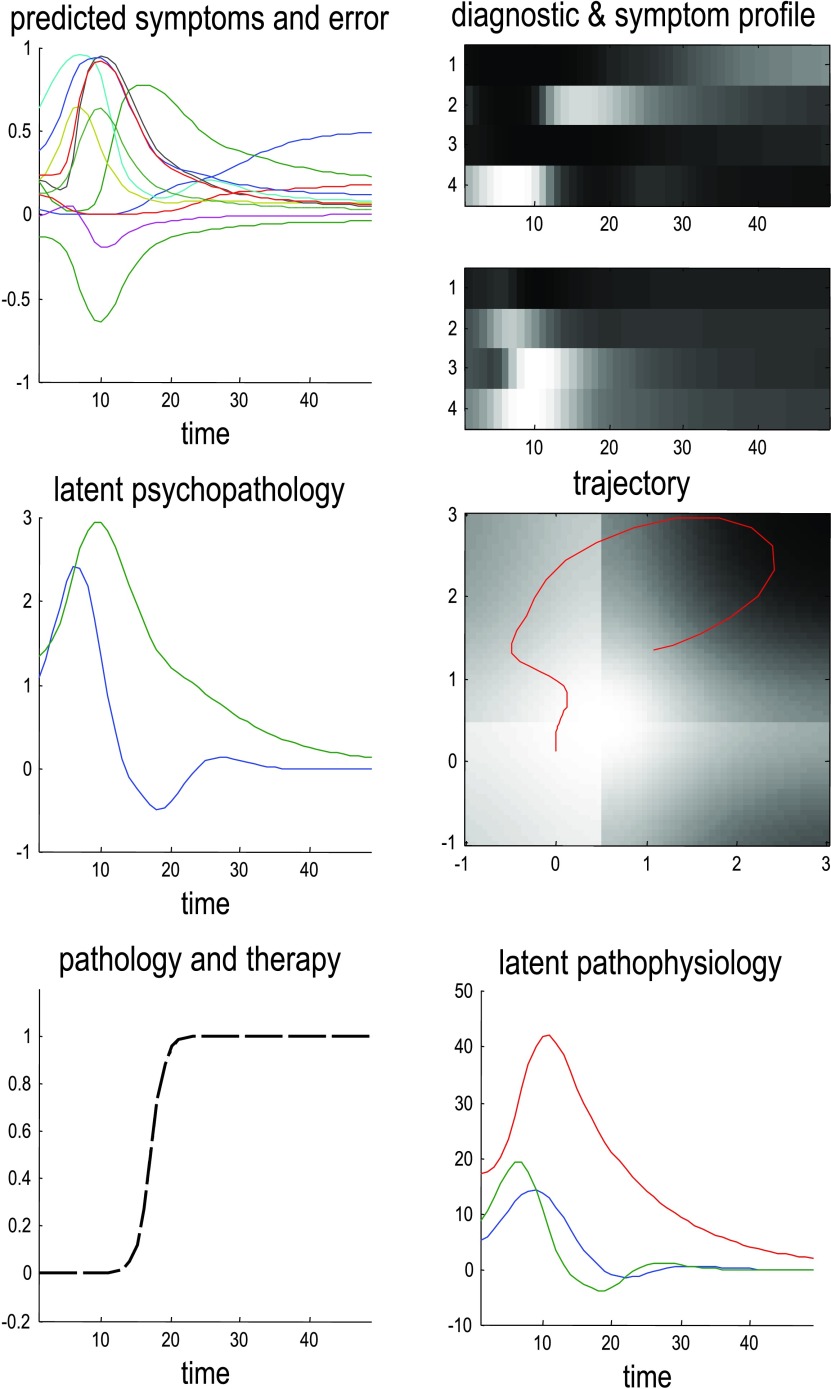
**Predicted responses to treatment.** This figure uses the same format as Figure 3; however, here the results are purely predictive in nature. In other words, the predictions are driven entirely by the pathophysiological dynamics based on subject-specific estimates of the model parameters—and starting from the state last estimated on the basis of an assessment prior to therapy.

**Figure 6.**
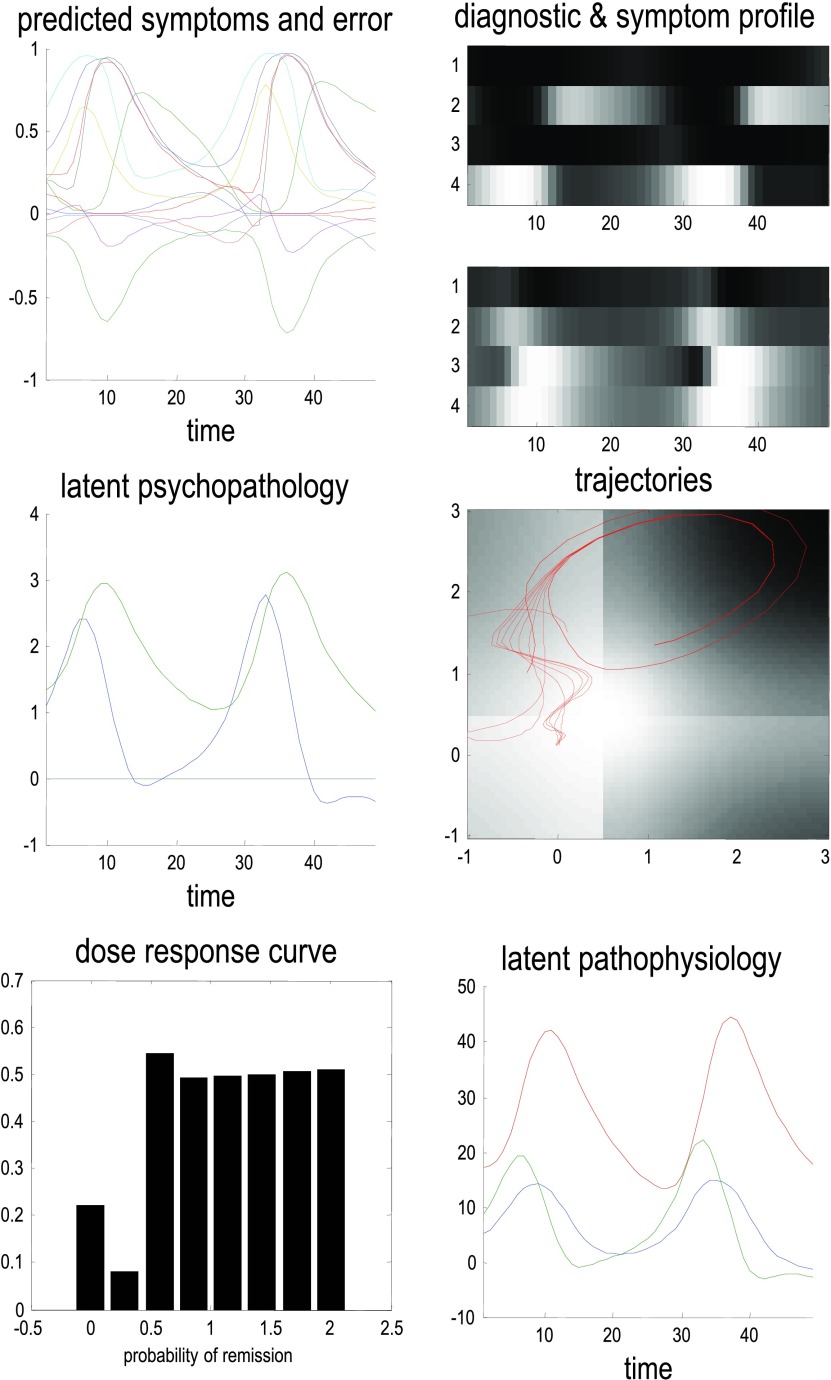
**Predicted responses to different treatments.** This figure uses the same format as the previous figure; however, here the results are shown in the absence of any treatment. The dotted trajectories in the state-space (middle right panel) report eight simulations over increasing levels of therapeutic intervention. The endpoints of these trajectories are summarized at the lower left in terms of the probabilities of receiving a diagnosis of “remitted.” This can be regarded as a predicted dose–response curve, illustrating the potential of the model to optimize treatment strategies.

Figure 5 shows the results of this predictive filtering using the same format as Figure 3. However, there are two crucial differences between Figures 5 and 3: First, we start from latent states that are posterior estimates of the subject’s current state, and more importantly, the trajectories are pure predictions based on pathophysiological dynamics. One can see that the predicted response to treatment (at 16 weeks) has an outcome similar to that of the actual treatment (although the trajectories are not exactly the same, when we compare the actual and predicted outcomes in Figures 3 and 5, respectively). Figure 6 shows the same predictions in the absence of treatment, again showing the pattern of fluctuation between schizophrenia and schizoaffective diagnoses that we encountered in Figure 2.

The middle right panel of Figure 6 also includes trajectories with increasing levels of therapeutic intervention (ranging from 0 to 2). The final outcomes of these interventions are summarized on the lower left in terms of the probabilities of a diagnosis of “remitted” at 48 weeks. This illustrates the potential for predictive modeling of this sort to provide dose–response relationships and explore different therapeutic interventions (and combinations of interventions). In this simple example, there is a small probability that the patient would remit without treatment, which dips and then recovers to levels around 50% with increasing levels of therapy. The apparent spontaneous recovery would, however, not be long-lasting—as can be imputed from the chaotic oscillations in Figure 1.

## CONCLUSION

We have illustrated what a computational nosology could look like by using simulations of clinical trajectories, under a canonical generative model. The potential of this approach to nosological constructs can be motivated from a number of perspectives. First, it resolves the dialectic between categorical diagnostic constructs (e.g., DSM) and those based on latent dimensions of psychopathology or pathophysiology (e.g., RDoC). Both constructs play essential roles in a generative modeling framework, as diagnostic outcomes and latent causes, respectively. To harness their complementary strengths, it is only necessary to determine how one construct follows from the other, which is an inherent objective of model inversion and selection. Second, we have tried to emphasize the potential for an evidence-based approach to nosology that can operationalize mechanistic hypotheses in terms of Bayesian model comparison. This provides an integration of basic research and clinical studies that could, in principle, contribute synergistically to an evidenced-based nosology. Finally, we have illustrated the practical utility of using the predictions of (optimized) generative models for individualized or precision psychiatry (Chekroud & Krystal, 2015), in terms of providing probabilistic predictions of responses to therapy.

A key aspect of the (diagnostic causal modeling) scheme above is that DSM/ICD diagnoses are treated as observations. This means that diagnoses become additional data that may provide further information (over and above symptoms) for model inversion. The advantage of this approach is that it provides a Bayes-optimal integration or fusion of multimodal (i.e., clinical and diagnostic) data. From a technical perspective, this integration is therefore grounded on the decades of careful feature selection provided by established diagnostic schemes. A potential disadvantage is that model inversion and comparison now essentially serve to identify those pathophysiological and psychopathological processes that best predict existing diagnostic categories, which may be heterogeneous in terms of an underlying pathophysiology. Having said this, heterogeneity is addressed implicitly by Bayesian model *reduction* and by the distinction between fixed and random (between-subjects) effects. As was noted by one of our reviewers, this offers a potentially “elegant approach, as it preserves the clinical intuition of an overarching syndrome and yet allows for inter-subject variability (at least as much as can be expressed through variations within the form of the chosen generative model).” However, it is not clear whether Bayesian model reduction will prove to be a sufficiently general solution. For example, it is possible that spectrum diseases consist of different subgroups or dimensions whose pathophysiology is described by qualitatively different models (i.e., nonnested or nonreducible likelihood functions). This speaks to an interesting application of Bayesian model *comparison* to provide evidence for—or against—qualitatively different pathophysiological variants that manifest as spectrum disorders.

### Relationship to Existing Frameworks

One might ask why dynamical systems predominate in our treatment. The answer rests upon the fact that neuronal dynamics, physiology, neuroendocrinology, and their behavioral (and autonomic) correlates are dynamic processes that can only be modeled with a dynamic or state-space model. In other words, clinical outcomes are generated by dynamic processes that necessarily call for a dynamic causal model. Any generative model that does not entail dynamics will, at best, only describe clusters (and associations) of symptoms (and signs). An emphasis on dynamic models is reflected in the modeling of infectious diseases (Paynter, 2016; White et al., 2007) and of immunological responses. Interestingly, these initiatives also have to contend with the balance between model complexity and accuracy (Thakar, Poss, Albert, Long, & Zhang, 2010)—supported by Bayesian model selection—and indeed with the very nature of causality (Vineis & Kriebel, 2006)—supported by dynamic causal models (Valdes-Sosa, Roebroeck, Daunizeau, & Friston, 2011).

It is interesting to contrast the computational nosology described here with the network analysis proposed in Borsboom and Cramer (2013). There are clear points of overlap, as well as some fundamental differences. Borsboom and Cramer use network analysis to dissolve some of the problems with traditional research strategies. They consider the utility of symptom networks, in which symptoms cause symptoms. The ensuing arguments about the ability to mimic symptom trajectories and model therapeutic interventions are compelling. Both the dynamic-causal-modeling and network analysis approaches can be regarded as responding to the limitations of current nosology, and both appeal to nonlinear, state-dependent interactions among the causes of symptoms. On the other hand, the notion that symptoms cause symptoms is antithetical to an underlying generative model—in which latent pathophysiology and psychopathology cause symptoms. In other words, if one subscribes to the approach described in this article, one commits to a formal distinction between measurable signs and symptoms (including clinical diagnoses) and the causes of those symptoms. For example, there is a fundamental distinction between a measurement (e.g., a temperature of 38.2 °C) and the causes of that measurement (e.g., bacterial infection). It is almost self-evident that to generate the (profile of) measurements available to a clinician, it is necessary to model their latent causes, whether or not they are ontologically well-defined.

Neuroimaging provides a cautionary tale here, which may usefully inform computational psychiatry. In neuroimaging, network analyses have been applied uncritically to measurements of hemodynamic or metabolic brain responses in order to furnish measures of *functional connectivity*. Although functional connectivity can be useful for phenotyping various individuals or cohorts, it does not provide insight into how the statistical dependencies among measurements are generated (Valdes-Sosa et al., 2011). To characterize the underlying network, one has to acknowledge that hemodynamic signals in one part of the brain do not cause hemodynamics in another, but rather are caused by—or are symptoms of—the underlying (latent) neuronal activity. Network analysis of the *effective connectivity* among neuronal systems (i.e., the causes) generating measurements (i.e., the consequences) almost invariably calls on some form of dynamic causal modeling (Moran, Symmonds, Stephan, Friston, & Dolan, 2011). This cautionary tale suggests that we should not look for correlations among symptoms. Rather, we should try to identify the causal (network) architecture among the symptoms’ latent causes: namely, the best generative model.

Both symptom network analysis (Borsboom & Cramer, 2013) and generative modeling eschew the common-cause framework—namely, the assumption that symptoms and signs can be uniquely attributed to a common cause. Indeed, the ill-posed nature of differential diagnosis can be particularly severe in psychiatry (e.g., Brendel & Stern, 2005). Any many-to-one mapping between latent causes and symptoms (and diagnostic constructs) can, in principle, be resolved through the Bayesian inversion of generative models that call on prior assumptions to resolve the implicit indeterminacy. The role of prior assumptions or constraints could, therefore, be regarded as finessing ill-posed problems that present beyond the common-cause framework.

### The Challenges of Generative Modeling

The choice of generative model is clearly crucial in terms of providing valid and useful predictions of treatment responses. In introducing dynamic causal models of pathophysiology and psychopathology, we have emphasized that the example used in this article is purely illustrative. So, how should one choose a generative model? Several issues make this choice simpler than it might appear at first glance. First, one does not have to choose a single model: The key benefit of Bayesian model comparison is that one can score the quality of different models in terms of their evidence—and thereby use strictly evidence-based criteria to select among plausible models. Second, at a phenomenological level, dynamic systems can be described in terms of a *normal form* (Murdock, 2003). In other words, some minimum description exists that preserves the essential dynamic behavior using a relatively simple set of differential equations. The notion of a normal form has proved extremely useful in the modeling of neu ronal dynamics—for example, in epilepsy (Roberts, Iyer, Finnigan, Vanhatalo, & Breakspear, 2014). This means that one does not need differential equations that describe every detailed fluctuation in the embodied brain; it is sufficient to summarize key fluctuations in terms of mixtures of physiological states with relatively simple differential equations (Freyer, Roberts, Ritter, & Breakspear, 2012). Finally, having established a normal form or minimal model, it is possible to successively refine the equations to move from a phenomenological to a more informed and mechanistic description. A nice example is the use of dynamic causal models in neuroimaging. These models started with a single, lumped neuronal state for each brain region—and a bilinear approximation to the underlying dynamics (Friston et al., 2003). These models were then progressively refined (through Bayesian model comparison and more informative data) to provide realistic descriptions of the synaptic activity and depolarization of specific neuronal populations (Moran et al., 2011). The hope here is that computational psychiatry could witness the same refinement of simple (e.g., normal-form) models over the same 5- to 10-year timescale seen in neuroimaging. The end result could be canonical models for particular illnesses, whose increasing complexity and neuroendocrinological realism is matched by an increase in the accuracy with which patient data are modeled in a continuous, medical setting. This long-term refinement is exemplified nicely by dynamic causal models of the mismatch negativity (Garrido, Kilner, Kiebel, & Friston, 2009) that now form the basis of routine applications—and that have found their way into psychiatric research (e.g., Ranlund et al., 2016).

Although we have emphasized the provisional nature of this approach, it should be acknowledged that one could analyze existing clinical data using the model described in this article with existing algorithms. Indeed, hundreds of publications in the neuroimaging literature have used dynamic causal modeling to infer the functional coupling among hidden neuronal states. In other words, it would be relatively simple to apply the techniques described above to existing data at the present time. However, the real challenge will lie in searching the vast model space to find models that are sufficiently comprehensive to account for the diverse range of clinical measures—in a way that generalizes from patient to patient—yet still parsimonious. This challenge is not necessarily insurmountable: One might argue that if we invested the same informatics resources in psychiatry that have been invested in weather forecasting and geophysical modeling, considerable progress could be made. Ultimately, one could imagine such model-based psychiatric prognoses being received with the same confidence that we currently accept daily weather forecasts. Of course, there are differences between psychiatric and meteorological forecasting. The latter has to deal with the “big data problem” in a relatively small model space. Conversely, psychiatry may have to contend with a “big theory problem” in a relatively large model space, but with more manageable datasets.

Having said this, perhaps the more important contribution of a formal nosology will not be its pragmatic application to precision medicine (i.e., through the introduction of prognostic apps for clinicians), but the use of Bayesian model selection to test increasingly mechanistic hypotheses and pursue a deeper understanding of pathogenesis in psychiatry. This is the way in which dynamic causal modeling has been applied in computational neuroscience and, as such, is just a formal operationalization of the scientific process.

## SOFTWARE NOTE

The simulations described in this article can be reproduced by downloading the SPM software (www.fil.ion.ucl.ac.uk/spm/) and running the MATLAB script DEM_demo_ontology.m. This demonstration uses the standard specification of dynamical systems used in the dynamic causal modeling of neurophysiological time series. For people who are interested in the technical or analytic aspects of model inversion, please see Friston et al. (2008) and Friston, Stephan, Li, and Daunizeau (2010) for a generalization of these schemes (see also https://en.wikipedia.org/wiki/Generalized_filtering). Their application in a variety of neuroimaging, cognitive, and computational neuroscience domains can be surveyed within the demo by using the graphical user interface opened by typing “DEM.” This provides access to annotated MATLAB scripts that offer a pseudocode specification of variational model inversion. For people interested in practical procedures, the simplest way to start would be to reproduce the inversion of fMRI time series described in the SPM tutorial (chap. 38), available from www.fil.ion.ucl.ac.uk/spm/doc/manual.pdf.

## ACKNOWLEDGMENTS

This article was prepared while J.A.G. was employed at Columbia University. The views expressed are his own and do not necessarily represent the views of the National Institutes of Health or the United States Government. K.J.F. is funded by the Wellcome Trust. We also thank our anonymous reviewers for helpful guidance in presenting these ideas. Karl J. Friston on behalf of the Ernst Strüngmann Forum on Computational Psychiatry: What Can Theoretical Neuroscience and Psychiatry Teach Each Other?

## Note

^1^ A *mixture* here is simply a linear or nonlinear combination of variables. Mixtures are therefore effectively patterns or modes over their constituent variables.
